# Learning structure of sensory inputs with synaptic plasticity leads to interference

**DOI:** 10.3389/fncom.2015.00103

**Published:** 2015-08-05

**Authors:** Joseph Chrol-Cannon, Yaochu Jin

**Affiliations:** Department of Computer Science, Faculty of Engineering and Physical Sciences, University of SurreyGuildford, UK

**Keywords:** synaptic plasticity, spiking neural networks, recurrent neural networks, inference, pattern recognition

## Abstract

Synaptic plasticity is often explored as a form of unsupervised adaptation in cortical microcircuits to learn the structure of complex sensory inputs and thereby improve performance of classification and prediction. The question of whether the specific structure of the input patterns is encoded in the structure of neural networks has been largely neglected. Existing studies that have analyzed input-specific structural adaptation have used simplified, synthetic inputs in contrast to complex and noisy patterns found in real-world sensory data. In this work, input-specific structural changes are analyzed for three empirically derived models of plasticity applied to three temporal sensory classification tasks that include complex, real-world visual and auditory data. Two forms of spike-timing dependent plasticity (STDP) and the Bienenstock-Cooper-Munro (BCM) plasticity rule are used to adapt the recurrent network structure during the training process before performance is tested on the pattern recognition tasks. It is shown that synaptic adaptation is highly sensitive to specific classes of input pattern. However, plasticity does not improve the performance on sensory pattern recognition tasks, partly due to synaptic interference between consecutively presented input samples. The changes in synaptic strength produced by one stimulus are reversed by the presentation of another, thus largely preventing input-specific synaptic changes from being retained in the structure of the network. To solve the problem of interference, we suggest that models of plasticity be extended to restrict neural activity and synaptic modification to a subset of the neural circuit, which is increasingly found to be the case in experimental neuroscience.

## 1. Introduction

Recurrent neural networks consisting of biologically based spiking neuron models have only recently been applied to real-world learning tasks under a framework called reservoir computing (Maass et al., 2002; Buonomano and Maass, 2009). The models of this framework use a recurrently connected set of neurons driven by an input signal to create a non-linear, high-dimensional temporal transformation of the input that is used by single layer perceptrons (Rosenblatt, 1958) to produce desired outputs. This restricts the training algorithms to a linear regression task, while still allowing the potential to work on temporal data in a non-linear fashion.

Given an initially generated static connectivity, reservoir computing is based on the principle of random projections of the input signal in which the network structure is completely independent of the input patterns. In these models, the only features learned by the trainable parameters of the perceptron readout are the correlations between the randomly projected features and the desired output signal.

We believe that learning in neural networks should go further than supervised training based on error from the output. All synapses should adapt to be able to encode the structure of the input signal and ideally, should not rely on the presence of a desired output signal from which to calculate an error with the actual output. The neural activity generated by the input signal should provide enough information for synapses to adapt and encode properties of the signal in the network structure. By applying unsupervised adaptation to the synapses in the form of biologically derived plasticity rules (Bienenstock et al., 1982; Bi and Poo, 1998; Wittenberg and Wang, 2006) it is hoped to provide the means for the recurrently connected neurons of the network to learn a structure that generates more effective features than a completely random projection that is not specific to the input data.

On a conceptual level, unsupervised learning is important in the understanding of how synaptic adaptation occurs because it is still unknown what the sources of supervised signals are in the brain, if any exist. From early work on synaptic self-organization (Hebb, 1949), the principle of learning has rested on correlations in neural activity becoming associated together and forming assemblies that activate simultaneously. These structures are thought to encode invariances in the sensory input that are key in developing the ability to recognize previously encountered patterns.

In this work we will explore the impact of applying several biologically derived plasticity mechanisms on three temporal sensory discrimination tasks. Two forms of spike-timing dependent plasticity (STDP) (Bi and Poo, 1998; Wittenberg and Wang, 2006) will be tested, along with the Beinenstock-Cooper-Munro (BCM) rule (Bienenstock et al., 1982). The sensory tasks will include real-world speech and video data of human motion. Synaptic plasticity will be applied in an unsupervised pre-training phase, before the supervised regression of the perceptron readout occurs. We will compare the impact that plasticity has on the performance in these tasks and also analyze the specific structural adaptation of the weight matrices between each of the classes of input sample in each task. A method will be introduced to evaluate the extent to which the synaptic changes encode class-specific features in the network structure.

Interference between different samples is a well-established phenomenon in sequentially trained learning models (McCloskey and Cohen, 1989; Ratcliff, 1990; French, 1999). When presented to a learning model, an input pattern will cause specific changes to be made in the models parameters—in the case of neural networks, the synapses. However, during this encoding process, existing structure in the synaptic values is interfered with. In this way, consecutive input patterns disrupt previously learned features, sometimes completely. This effect is known as forgetting. It is of direct concern to neural networks trained on sensory recognition tasks that consist of spatio-temporal patterns projected through a common neural processing pathway. We will quantify the level of interference between the synaptic parameters for each tested plasticity model being applied to each type of sensory data.

Existing studies report that adapting neural circuits with plasticity improves their performance on pattern recognition tasks (Yin et al., 2012; Xue et al., 2013) but there is no analysis of how the adaptation of synaptic parameters leads to this result. On the other hand, work that does detailed analysis on the structural adaptation of the network does so using synthetic input patterns that are already linearly separable (Toutounji and Pipa, 2014) or Poisson inputs projecting to single and recurrently connected neurons (Gilson et al., 2010). For a review of work applying plasticity models to improve the general properties of neural networks, the reader is referred to Chrol-Cannon and Jin (2014a).

The experiments undertaken in this work will be performed on a typical reservoir computing model with its recurrent connections adapted with plasticity. Two main angles of analysis are made; we determine the strength of input specific synaptic adaptation and the extent to which consecutive inputs interfere within the synapses. Both of these are achieved through analysis of the change in weight matrix in response to each pattern.

## 2. Results

### 2.1. Training recurrent networks with plasticity

Our training and analysis is performed on a typical liquid state machine (LSM) model (Maass et al., 2002) that is trained to correctly classify temporal input patterns of sensory signals. Details of the models and simulations can be found in the Section 4. Here we present an overview of the experimental procedure.

An LSM consists of recurrently connected spiking neurons in which transient activity of the neurons is driven by time-series input sequentially exciting their membrane potential. In order for an output to be produced from the network and used to train a supervised readout, a snapshot must be taken of the transient activity which we call the state vector. This vector is weighted and summed to produce an output, the weights of which are trained with linear regression.

In our experiments we adapt the recurrent connections with synaptic plasticity before taking the state vectors used for pattern recognition. We intend to change the synaptic weights from their initial random structure, to values that are adapted to the general statistics of the input signals. After this pre-training process, we take the state vectors for each sample in the data set and use it to train a set of readouts to recognize labeled patterns in the data. Performance of pattern recognition is only a small aspect of our analysis of synaptic adaptation through plasticity. The analysis methodology described in the next subsection requires the information of how each sample of input causes unique adaptation of the synapses. Therefore, for convenience, when collecting the liquid state vectors of a given sample from the neural activity, we also compute the synaptic change during the presentation of that sample and store the weight matrix adaptation.

**Figure 1:**
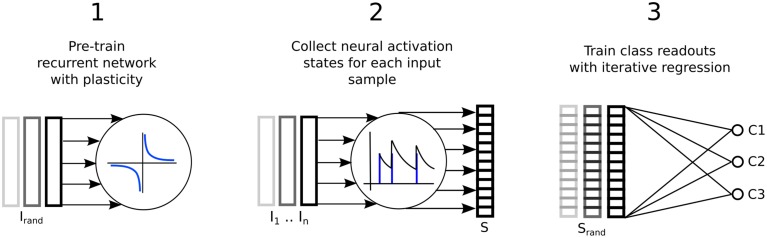
**Three step process describing a reservoir computing model extended by having the recurrent connections adapted with unsupervised plasticity in a pre-training phase**. Firstly, input samples *I* are presented in random order while the resulting neural activity drives synaptic adaptation under plasticity. Secondly, each input sample is presented in sequence with the resulting neural activity decoded into a series of state vectors *S*. Finally, the state vectors are used as the input to train a set of perceptron readouts, one to recognize each class of sample, *Cx*.

Figure 1 illustrates the three step process just described, delineated into; a pre-training phase of synaptic plasticity, a collection of the liquid state vectors and weight adaptation matrices, and a supervised training phase of linear readouts for pattern recognition.

### 2.2. Description of sensory inputs

Complex sensory signals are projected through a common set of nerve fibers to cortical regions that must learn to distinguish between them based on differences in their spatial-temporal features.

Three sensory recognition tasks are selected, among which two of them consist of real audio and video signals of human speech and motion. For all tasks, the neural network output is trained to respond uniquely to each of the different types of input sample and therefore be able to perform effective recognition between them. Also, sample specific synaptic adaptations are analyzed to determine if unique structure is learned within the network due to synaptic plasticity.

**Figure 2:**
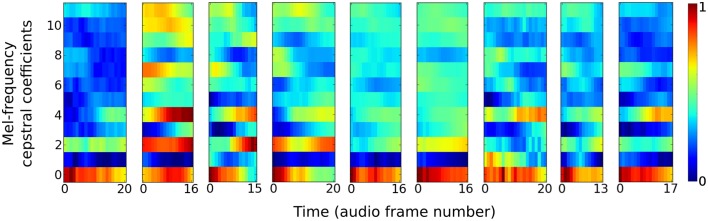
**Vowel samples from the nine speakers in the speaker recognition task**. The audio signals in the data set are pre-processed into 12 Mel-frequency cepstrum coefficients (MFCC) features. Samples from each speaker have variable time duration in the number of audio frames they consist of.

The auditory task is to distinguish between nine different speakers based on short utterances of the vowel **/ae/**. Each of the 640 samples consists of a frequency “spectrogram” that plots frequency intensity over a sequence of audio time frames. Figure 2 plots an example sample from each of the nine speakers.

**Figure 3:**
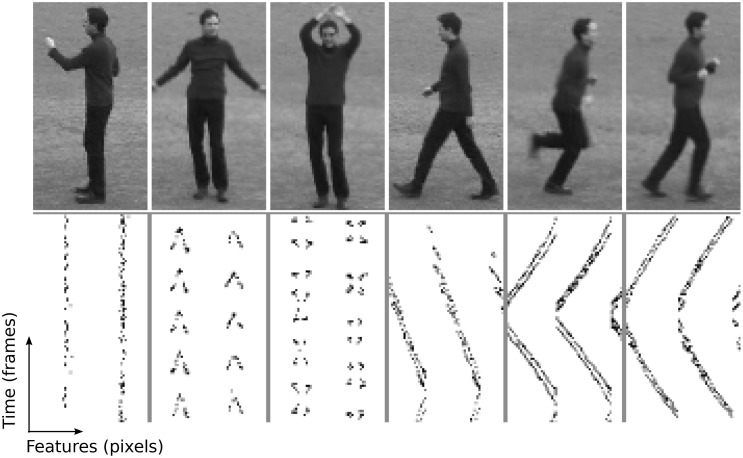
**Human motion samples for the six types of behavior in the KTH visual discrimination task**. This illustration consists of different behaviors from a single person, while the whole data set contains 25 persons. **Top row:** Still frames from example video samples; boxing, clapping, waving, walking, running and jogging. **Bottom row:** Features extracted corresponding to the samples above, according to Equations (13) and (14). Features are the raw time-series activity used as input to the neural network.

The visual task is to distinguish between six types of human behavior; boxing, clapping, waving, walking, running and jogging. The 2391 samples are video sequences of many different subjects performing those six motions. There is a simple pre-processing stage that converts the video data into a sparse representation before being used as input to the neural network. Extracted still frames and processed features are plotted in Figure 3 for one subject performing each of the six behaviors.

**Figure 4:**
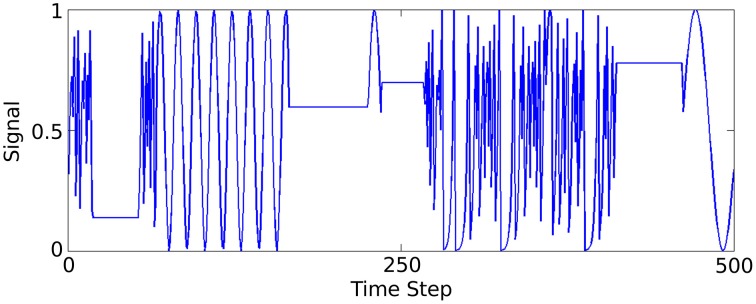
**Plot of 500 of the 50,000 data samples generated according to Jaeger's tri-function system recognition time-series task (Jaeger, 2007)**.

A synthetic data set is generated to model a low spatial dimension but very high frequency temporal structure, in contrast to the previous two sensory tasks. Three functions generate time-varying single dimensional signals that the network learns to distinguish between. A complete description and method for generating the data is described in Jaeger (2007) (Figure 4) illustrates part of this signal.

The auditory and visual tasks are described in Kudo et al. (1999) and Schuldt et al. (2004), respectively, with data availability also provided.

### 2.3. Analysis of synaptic adaptation

**Figure 5:**
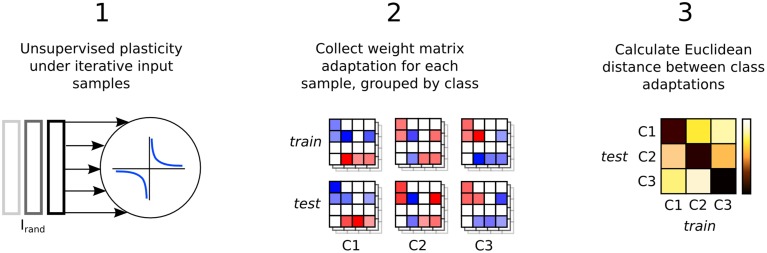
**Three step process describing the analysis of input-specific synaptic adaptations**. Firstly, the recurrent connections are adapted under plasticity in the same way as in Figure 1. Secondly, each input sample is presented and plasticity adapts the synapses. The change in the weight matrix is stored for each sample and grouped by the input class label, *Cx* and into two sets, *train* and *test*. Finally, the Euclidean distance between the matrices in *train* and *test* is calculated and the average for each class label is plotted in a confusion matrix.

Synaptic weight adaptation matrices form the basis of the analysis in this work. Figure 5 depicts the process of these matrices being collected and used for analysis of class-specific synaptic plasticity. Firstly, synaptic plasticity is applied to the network to adapt a baseline weight matrix that reflects the general statistics of the input patterns in the data set. Secondly, each the weight adaptation matrix is collected for each sample and these are grouped by class and also into two sets based on the training and testing data division. Finally, the Euclidean distance is calculated between each weight matrix, with the average distance between each set plotted in a type of “confusion matrix” in which a low distance indicates high similarity between the adaptation of synaptic parameters.

In the confusion matrix just described, if the diagonal values are lower than the others it means that synaptic plasticity is sensitive to the structural differences in input samples that are labeled as different classes. The stronger the diagonal trend, the more sensitive plasticity is to features of the input. It means that plasticity learns to distinguish class labels, such as different speakers or human actions, without ever being exposed to the labels themselves *a priori*.

The weight adaptation matrices are also used to estimate the amount of interference between different input samples within the synaptic parameters. This is described further later in the Results Section.

### 2.4. Learning input-specific adaptations using plasticity

We wish to test the hypothesis that synaptic plasticity is encoding a distinct structure for input samples of different labels. For the speech task, these labels consist of different speakers and for the video recognition task the labels consist of different human behaviors.

The data sets are divided evenly into two. Each subset is used to train a recurrently connected network for 10,000 iterations, selecting a sample at random on each iteration. The changes to the weight matrix due to plasticity are recorded for each sample presentation. This is then used to create a class-specific average weight change for each of the class labels in both of the sample subsets. Finally, we calculate the Euclidean distance between each class in one set and each class in the other according to the following formula:

(1) $$\begin{array}{l} \mathrm{Dist}\left(C_{lab}^{X},C_{lab}^{Y}\right)=\overset{N}{\underset{i=1}{\sum }}|\Delta W_{i}\left(C_{lab}^{X}\right)-\Delta W_{i}\left(C_{lab}^{Y}\right)| \end{array}$$

Where *C*_*lab*_ denote class labels, *X* and *Y* distinguish the separated sets of samples, Δ*W* is the change in weight matrix for a presented sample, *N* is the number of synapses, and *i* the synapse index.

This effectively produces a confusion matrix of similarity in the synaptic weight change for different classes of input. Having lower values on the descending diagonal means that there is structural adaptation that is specific to the class of that column compared with the similarity between structural adaptations of two different classes.

**Figure 6:**
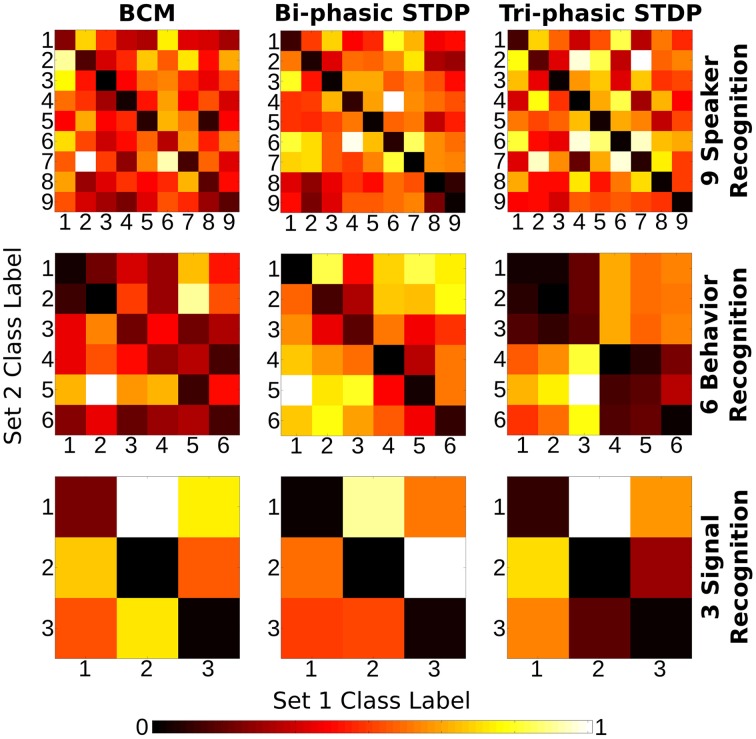
**Class correlation of structural synaptic adaptation**. Heat map plots indicate the structure learned on each class for the three tasks under each of the plasticity rules. Essentially, it is a confusion matrix of the geometric distance between the weight matrix adaptation of each class of sample. The training data for each task is divided into two sets. Class-average adaptation is found for each set. There is then a distance calculated between each class of the two sets. Lower values on the descending diagonal indicate higher correlation within a class adaptation and therefore strong class-specific structure learned.

Figure 6 shows the “weight change confusion matrices” described above, for each plasticity model applied to all sensory tasks (nine experiments in total). All of the experiments show at least some stronger similarity in the descending diagonals and most are stark in this manner. It is certainly a strong enough pattern to show that through the many iterations of training, each of the plasticity models have become sensitive to the particular structure of the sensory input signals so that each different class of sample will give rise to changes in synaptic strength that are distinct from other classes compared with the similarity to themselves. We re-iterate that the class labels were not used in any way in the plasticity models themselves and so the differences in the weight change arise from the input signals alone.

There are a few exceptions to the strong diagonal patterns in Figure 6. This means that some classes are not effectively distinguished from each other; speakers 8/9 with bi-phasic STDP, behaviors 1/2 with BCM, behaviors 1/2/3, and 4/5/6 with tri-phasic STDP. The latter confusion corresponds to the behaviors of boxing/clapping/waving and walking/running/jogging. From the similarity of those input features shown in the lower panes of Figure 3, it is evident why this confusion might occur.

### 2.5. Classification performance with plasticity

Perhaps the ultimate goal of neural network methods when applied to sensory tasks is the ability to accurately distinguish different types of input sample by their patterns. We compare the error rates achieved by our neural network on the three sensory tasks, with and without the different forms of plasticity used in this work. Table 1 lists the error rates achieved for each of the learning tasks with the different plasticity rules active in a pre-training phase in addition to a static network with fixed internal synapses.

**Table 1:** **Classification error rates**.

	**Static**	**BCM**	**STDP**	**TP-STDP**
Tri-func	0.153	0.157	0.204	**0.138**
KTH	**0.283**	0.3	0.333	0.383
Vowels	0.089	**0.086**	0.092	**0.086**

*Values averaged over 10 trials with random seed based on system clock. SD did not exceed 0.03 for all values. Bold values indicate lowest error rate*.

From the error rates in Table 1 it is evident that pre-training the network with synaptic plasticity can make insignificant improvements in lowering the error rate. However, the results here indicate that it can have a greater negative impact than a positive one. In the KTH human behavior data set, all three plasticity models increase the error rate by between 1.7 and 10%. Conversely, the best improvement was found on the tri-function signal recognition task with tri-phasic STDP at only 1.5%.

It is clear from the network output that pre-training with synaptic plasticity is not a suitable method for this class of model, This does not contradict the result that plastic synapses are learning useful, input-specific structure. However, it does suggest that the structure being learned is not effectively utilized in the generation of a network output. We next investigate interference between synaptic changes to determine if the structural learning is retained in the network or if interference is a barrier for effective application of synaptic plasticity.

### 2.6. Synaptic interference

When a model adapts incrementally to sequentially presented input, existing patterns that have been learned by the model parameters are prone to be overwritten by learning new patterns. This is known as interference. The work that has studied this effect (McCloskey and Cohen, 1989; Ratcliff, 1990; French, 1999), test the ability to recognize previously presented input after the model has been trained on new ones in order to estimate how much learning has been undone. When new training leaves the model unable to recognize old patterns, it is said there has been catastrophic interference and forgetting.

We introduce a method of measuring interference directly in synaptic parameters instead of the model output. Our measure is described in detail in the Section 4. *I*^*total*^ directly quantifies all synaptic changes that are overwritten.

The interference for each of our experiments is listed in Table 2. In all but one of the experiments the interference level is between 82 and 96%. Most of the learned structure for each class of input is forgotten as consecutive samples overwrite each other's previous changes. Bi-phasic STDP applied to speaker recognition has the lowest level of interference at 58%.

**Table 2:** **Synaptic interference**.

	**BCM**	**STDP**	**TP-STDP**
Tri-func	0.82	**0.8**	0.88
KTH	**0.92**	0.93	0.96
Vowels	0.96	**0.58**	0.9

*Values averaged over 10 trials with random seed based on system clock. SD did not exceed 0.07 for all values. Bold values indicate lowest interference*.

**Figure 7:**
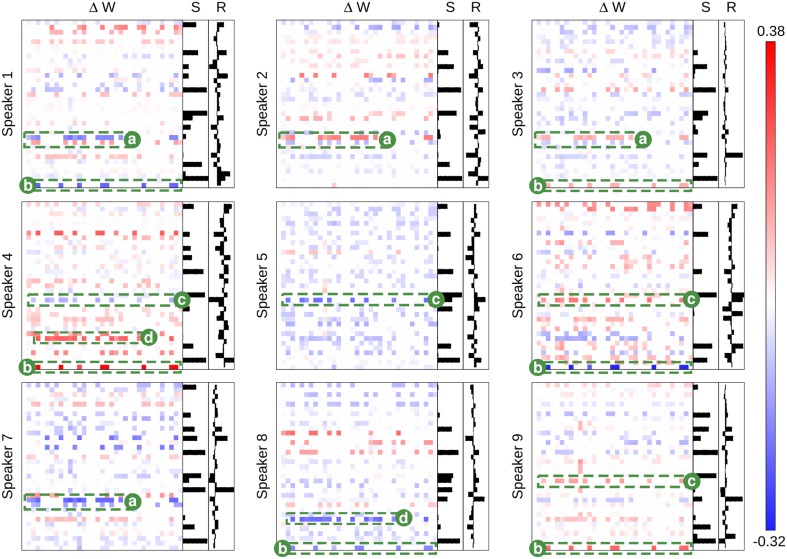
**The class-specific synaptic adaptation for the 9 class speaker recognition task under BCM plasticity**. The main heat maps in each subplot show the adaptation of the weight matrix (synapses) after the presentation of voice input data from each speaker. Blue values show a reduction in synaptic strength and red values show an increase. Each N × N weight matrix has *pre-neurons on the x-axis* and *post-neurons on the y-axis*. The bar-chart, **S**, shows the average neuron activation for each class. The bar-chart,**R**, shows the learned readout weights. Labeled synapses **a–d** indicate key structural changes that are selective between different speakers. Each label alone can distinguish between two sets of speaker. Taken all together, the labeled synapses adapt specifically to each speaker in a unique pattern, learning a distinct network structure for each one.

To further explore interference and visualize the impact of plasticity, synaptic changes will be analyzed directly. Figure 7 is an illustrative example in which a reduced network size of 35 neurons is used to improve visual clarity of the plotted patterns. It is an example for the speaker recognition task with BCM plasticity with similar figures for the other experiments given in Supplementary Figures 1–8. It shows the adaptation of the synaptic weight matrix produced by each speaker in the voice recognition task. This is plotted against the activity level for each neuron, **S**, and the readout weights, **R**, that are trained to generate an output that is sensitive to that given speaker. Each of these sub plots is the average response taken over all sample presentations from that speaker. This makes a whole chain of effect visible: from the synaptic change of an internal network connection, to the average neuron state for a given speaker, to the selective weights of the readout for that speaker. For all to be working well in a cohesive system, we expect that a positive weight change should correspond with a neuron activation unique to the class which would in turn improve the recognition ability of the readout to identify that class.

The sections of the class weight matrix highlighted in green in Figure 7, highlight an example where synaptic interference is occurring between different types of pattern. Directly opposing features in the weight matrix adaptations show the samples negating each other's changes. However, the same features are also most distinctively class specific.

Any synapse can only change in two directions: positively or negatively, which means that a single synapse can only adapt to distinguish between two mutually exclusive kinds of input pattern. If *n* synapses are considered in combination, then the number of input patterns that can be discriminated becomes 2^*n*^ in ideal theoretical conditions. Figure 7 illustrates this principle in practice with regards to the nine speaker recognition tasks. The adapted synapses labeled (a) can clearly distinguish speaker {#1} from speakers {#2, #3} but cannot distinguish {#2} from {#3}. Similarly, the adapted synapses labeled (b) can distinguish speakers {#1, #6, #8} from speakers {#3, #4, #9} but cannot distinguish speakers within either of those sets. However, if the synapses (a–d) are considered in combination, then all speakers can be distinguished by synaptic plasticity changes alone.

Figure 7 also shows the weight changes are not correlated with the neural activity or readout weights. For plasticity to improve the accuracy of sensory discrimination, it would be expected that synapses would strengthen for class specific neural activity and weaken for common neural activity. This is not the case in our results.

## 3. Discussion

### 3.1. Evolution of synaptic weights

Our main conclusions are drawn from the observation that the synaptic plasticity models tested become sensitive to specific class labels during a competitive process of synaptic interference between input patterns. For our conclusions to be generally applicable to recurrent neural circuits and liquid state machines in particular, we must demonstrate that synaptic weights reach some stability during pre-training and that the neural activity dynamics are working in a balanced regime.

**Figure 8:**
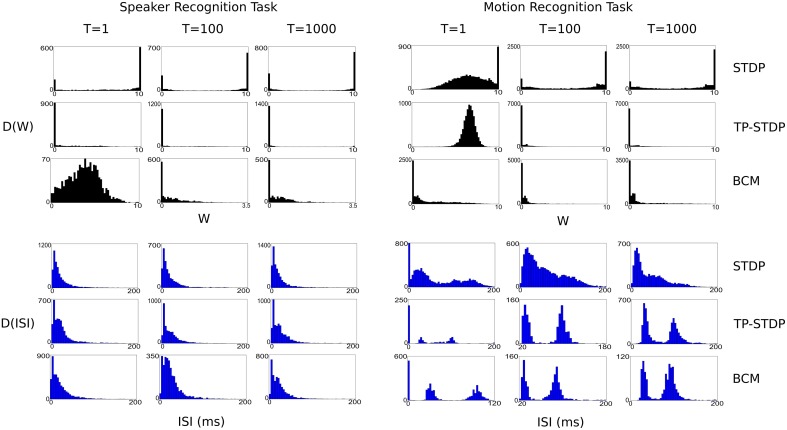
**Plots of evolving synaptic weight (black, top) and ISI (blue, bottom) distributions given for each recognition task and each plasticity model**. The plots are snapshots of the parameter distributions after 1, 100, and 1000 input samples have been presented during pre-training.

Figure 8 shows a series of plots taken at 1, 100, and 1000 input iterations that show the evolving distributions of synaptic weights and inter-spike intervals (ISI) for each of the experiments performed in this work.

In general, the plots show that between the first and 100th pattern, the synaptic weights are adapted significantly by plasticity, with a corresponding—but more subtle—change in the distribution of ISIs. While there is also some level of change in weights between the 100th and 1000th iteration, the level is far smaller, which indicates that the synapses are converging on a common structure. However, it is important to note that for simulations even up to 10,000 iterations there is always some low level of synaptic change. The plasticity models tested never stabilize to a point in which there is no further synaptic adaptation, even when we repeatedly present a single input sample.

Each of the plasticity models drives the synaptic weights to a different kind of distribution. STDP creates a bi-modal distribution that drives most weights to the extremes: 0 and 10, with a few that are in a state of change leading up to each boundary. It leads to a structure with more full strength synapses than zeroed. TP-STDP and BCM plasticity leads to sparser connectivity that drives most weights to zero. In particular, TP-STDP only maintains a small number of weak connections due to the narrow window of potentiation being surrounded by depressive regions that suppress most connections. BCM includes an implicit target level of post-synaptic activity that encourages some synapses to take larger values but doesn't drive them to their maximum.

The distribution of ISIs give an indication of the dynamics of the neural activity. The plots in Figure 8 show that a balance between completely sparse and saturated activity is maintained during the simulation. The shape of the ISI distributions tend to stabilize between 100 and 1000 sample presentations.

The above observations provide some evidence that the results presented in this article are not simply an artifact of a particular choice of model parameters but are observed for a normally functioning liquid state machine.

### 3.2. Unsupervised plasticity learns label specific structure

Both STDP and BCM models adapt the synapses of a network in distinctive patterns according to which type of sample is being presented to the network. We can conclude that presenting a training signal with the sample label is not required for plasticity to learn specific information for complex sensory inputs from different sources. This result holds for the speech, visual and benchmark pattern recognition tasks. To achieve this feat, we hypothesize that plasticity drives the synaptic parameters to a structure that represents an average between all input samples. Once converged, any further input stimulus will drive the synaptic parameters in a unique direction away from this average structure. On balance, scrambled presentation of random inputs keeps the network in this sensitive state.

### 3.3. Uniformly applied plasticity leads to synaptic interference

We show synaptic plasticity spends most of its action counter- acting previous changes and overwriting learned patterns. The same patterns of synaptic adaptation that distinctly characterizes each class of input are the same ones that reverse adaptations made by other inputs.

Plasticity is applied uniformly to all synapses. All neurons in a recurrent network produce activity when given input stimulus. Combined, these factors mean that any input sample will cause the same synapses to change. This leads to synaptic competition, interference and ultimately, forgetting.

### 3.4. Local plasticity required to overcome interference

To overcome the problem of interference, the mechanisms of plasticity need to be restricted to adapt only a subset of the synapses for any given input stimulus. There is much existing research that supports this conclusion and a number of possible mechanisms that can restrict the locality of plasticity.

It has been shown *in vivo* (using fMRI and neurological experiment) that synaptic plasticity learns highly specific adaptations early in the visual perceptual pathway (Karni and Sagi, 1991; Schwartz et al., 2002). Simulated models of sensory systems have demonstrated that sparsity of activity is essential for sensitivity to input-specific features (Finelli et al., 2008; Barranca et al., 2014). In fact, in a single-layer, non-recurrent structure, STDP is shown to promote sparsity in a model olfactory system (Finelli et al., 2008). Conversely, in recurrent networks, STDP alone is unable to learn input specific structure because it “over-associates” (Bourjaily and Miller, 2011). Strengthened inhibition was used to overcome this problem and combined with reinforcement learning to produce selectivity in the output (Bourjaily and Miller, 2011). By promoting sparsity, the lack of activity in most of the network will prevent activity-dependent models of plasticity in adapting those connections.

Reward modulated plasticity has also been widely explored in simulated (Gavornik et al., 2009; Darshan et al., 2014) and biological experiment (Li et al., 2013; Lepousez et al., 2014). Input-specific synaptic changes are shown to be strongest in the presence of a reward signal (Gavornik et al., 2009; Lepousez et al., 2014). Lasting memories (synaptic changes not subject to interference), are also seen to rely on a process of re-consolidation consisting of fear conditioning (Li et al., 2013). A reinforcement signal based on either reward or fear conditioning can be effectively used to restrict synaptic changes in a task dependent context such as sensory pattern recognition.

Another way to restrict synaptic changes in a task dependent way is to rely on a back-propagated error signal that has well-established use in artificial neural networks. This might be achieved in a biologically plausible way through axonal propagation (Kempter et al., 2001) or top-down cortical projections sending signals backwards through the sensory pathways (Schäfer et al., 2007). Top-down neural function in general is thought to be essential in determining structure in neural networks (Sharpee, 2014), providing a context for any adaptations. A molecular mechanism for the retro-axon signals required for back-propagation is has been proposed (Harris, 2008). However, in general these retro-axon signals are known to be important for neural development but may be too slowly acting to learn sensory input.

### 3.5. Learning input structure does not necessarily improve performance

Structural adaptation with plasticity in the pre-training phase, while specific, may not be utilized by the output produced by the network readout. This could be due to the following reasons. Firstly, there is a disparity in the neural code. The output from a recurrent spiking network model is currently decoded as a rate code. In contrast, synaptic plasticity updates structure in a way that depends on the precise temporal activity of neural spikes. Secondly, information content is reduced. While creating associations between co-activating neurons, Hebbian forms of plasticity may also increase correlations and reduce information and separation. These can determine the computational capacity of a recurrent network model (Chrol-Cannon and Jin, 2014b). Both discrepancies could be barriers for the effective application of plasticity to improve pattern recognition. Therefore, new frameworks of neural processing should be based directly on the adapting synapses. This will lead to functional models of neural computing that are not merely improved by synaptic plasticity, but that rely on it as an integral element.

This finding contrasts with some existing work that shows pre-training with plasticity including STDP (Xue et al., 2013) and BCM (Yin et al., 2012) can improve performance in a recurrent spiking network. To address this discrepancy we note that pre-training might improve the general computational properties of recurrent networks without learning input-specific structure. Furthermore, if this is the case, the likelihood of plasticity leading to an improvement will largely depend on how well-tuned the initial parameters of the network are before the pre-training phase begins.

## 4. Materials and methods

### 4.1. Simulation procedure

The three step procedure depicted in Figure 1 for training an LSM with plasticity is now described below in pseudocode. Where relevant, some of the expressions within the pseudocode refer to equations that can be found in subsequent subsections where the models for neurons, connectivity, plasticity and pre-processing of inputs can also be found.

Firstly, the following section of pseudocode illustrates the pre-training process in which the recurrent synaptic connections are adapted with plasticity. Input samples are selected at random (scrambled) for a total number of *preTrainIterations* which is 10,000. For a single input sample, each of the time-series frames is presented to the network in sequence by setting the input current of the connected neurons to *W*_*in*_[*x*][*c*]·*S*[*f*][*x*]·*inputScale*. The *inputScale* is 20, which is based on the neuron membrane model selected. The neural activity of the network is then simulated for *frameDuration* which is 30 ms. Plasticity is calculated and updated in between each frame of input in a sample. Neural activity is reset for the next input sample.


// pre-train recurrent neurons with
                                   *plasticity*
  **for each** iteration *I* **in** *preTrainIterations*
      select random sample *S* from *trainingSamples*
      **for each** frame *f* **in** *S*
          **for each** attribute *x* **in** *f*
              **for each** connection *c* **in** *C_in_*
                  *c*.input(*W_in_*[*x*][*c*] · *S*[*f*][*x*] ·
                                     *inputScale*)
          **for each** timestep *t* **in** *frameDuration*
              *neurons*.simulateActivity()
                        // *Equations  2, 3, 4*
          *synapses*.applyPlasticity()
                        // *Equations 8, 9, 10*
      *neurons*.resetActivity()


Secondly, we collect the reservoir states for each sample. The simulation procedure is essentially the same as in pre-training but iterates once for each sample in the dataset. Activity feature vectors are stored in *S*.*fv* and weight matrix adaptation in *S*.*dw*.


 // *collect neural activation state vectors*
  *baseWeights.value* ← *synapses.value*
  **for each** sample *S* **in** *trainingSamples*
      **for each** frame *f* **in** *S*
          **for each** attribute *x* **in** *f*
              **for each** connection *c* **in** *C_in_*
                  *c*.input(*W_in_*[*x*][*c*] · *S*[*f*][*x*] ·
                                     *inputScale*)
          **for each** timestep *t* **in** *frameDuration*
              *neurons*.simulateActivity()
                         // *Equations 2, 3, 4*
          *synapses*.applyPlasticity()
                        // *Equations 8, 9, 10*
      *S.fv* ← *neurons*.filteredSpikes()
                                // Equation 5
      *S.dw* ← *synapses.value* - *baseWeights.value*
      *neurons*.resetActivity()
      *synapses.value* ← *baseWeights.value*


Finally, for determining the pattern recognition performance of the LSM, we train a set of readouts using least mean squares regression. There is one readout to predict the presence of each possible class of input. For a total of *readoutTrainingIterations* that is set to 100,000, a randomly selected samples state vector *fv* will be used to adapt the readout weights. The desired signal will be set to 1 for the readout matching the sample class and 0 for the others. For predicting class labels on the training and testing data, the readout with the maximum value for a given *fv* is selected to predict the class (winner takes all).


  // *train readouts with linear regression*
  **for each** iteration *I* **in** *readoutTrainIterations*
      select random feature vector *fv* from
                      *trainingSamples.fv*
      **for each** class readout *R* **in** *nClass*
          **if** *R.classLabel* = *fv.classLabel*
              // *boost readout for matching*
                                        *class*
              *R.output* ← *R.lms*(*fv*, *1*)
                           // *Equations 6, 7*
          **else**
              // *suppress other readouts*
              *R.output* ← *R.lms*(*fv*, *0*)
                           // *Equations 6, 7*
      prediction *P* ← *max*(*R.output*)
      **if** *P.classLabel* ≠ *fv.classLabel*
          *errorSum* ← *errorSum* + *1*
      *errorCummulative* ← *errorSum* ÷ *I*


### 4.2. Recurrent network

**Figure 9:**
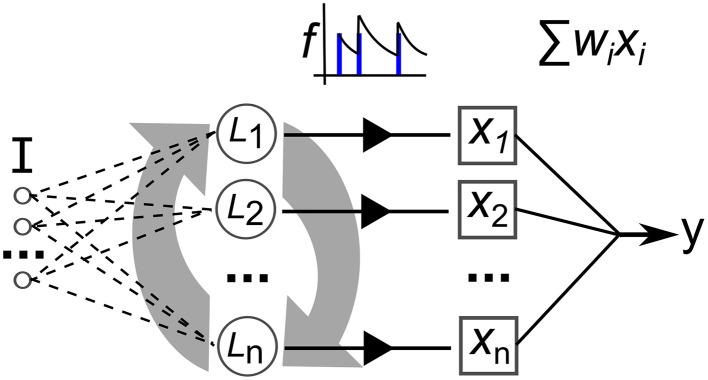
**Depiction of the elements of our recurrent network model**. ***I*** is a multi-dimensional input signal, ***L*** nodes constitute the recurrent network, the ***x*** vector is the neural activation state, ***f*** is the filtering of the spike trains and ***y*** is the output after weight and sum.

The neural network model used in this work is illustrated in Figure 9. Recurrently connected neurons, indicated by *L* are stimulated by current *I* that is the sum total of injected current from the input signal, *I*_*inj*_ and stimulating current from the pre-synapses, *I*_*rec*_. The total current *I* perturbs the membrane potential that is modeled with a simple model that matches neuron spiking patterns observed in biology (Izhikevich, 2003). This method for modeling the spiking activity of a neuron is shown to reproduce most naturally occurring patterns of activity (Izhikevich, 2004). The real-valued inputs are normalized between 0 and 1, which are multiplied by a scaling factor of 20 before being injected as current into *L*. Input connections number 0.2·*networksize*, projected randomly to the network nodes. Weights are uniformly initialized at random between 0 and 1. The video data set used in this work consists of significantly higher dimension inputs—768 features—than the other data sets. Therefore, in this case each feature only projects to one neuron, initially selected at random (a neuron can have connections from multiple inputs). Also the synaptic weights are scaled by 0.25.

The network activity dynamics are simulated for 30 ms for each frame of data in a time-series input sample. This value is chosen as it roughly approximates the actual millisecond delay between digital audio and video data frames. Then, the resulting spike trains produced by each of the neurons are passed through a low-pass filter, *f*, to produce a real valued vector used to train a linear readout with the iterative, stochastic gradient descent method (each described in the next section).

In our experiments the network consists of 35 or 135 spiking neurons (weight matrix plots consist of 35, performance trials consist of 135) with the ratio of excitatory to inhibitory as 4:1. Neurons are connected with static synapses i.e., the delta impulse (step) function. Connectivity is formed by having *N*^2^ · *C* synapses that each have source and target neurons drawn according to uniform random distribution, where *N* is the number of neurons and *C* is 0.1, the probability of a connection between any two neurons. Weights are drawn from two Gaussian distributions; 𝓝(6, 0.5) for excitatory and 𝓝(−5, 0.5) for inhibitory. When plasticity adapts the reservoir weights, *w*_*max*_ is clamped at 10 and *w*_*min*_ at −10. All parameters for excitatory and inhibitory neuron membranes are taken from Izhikevich (2003). The equations for the membrane model are as follows:

(2) $$\begin{array}{rcl} v^{\prime } & = & 0.04v^{2}+5v+140-u+I \end{array}$$

(3) $$\begin{array}{rcl} u^{\prime } & = & a\left(bv-u\right) \end{array}$$

With the spike firing condition:

(4) $$\text{if }v>30mV\text{ then }\{\begin{array}{l} v\leftarrow c\\ u\leftarrow u+d \end{array}$$

Parameters for the above equations are; *a* = 0.2, *b* = 0.2, *c* = −65, *d* = 8 for excitatory neurons and; *a* = 0.1, *b* = 0.2, *c* = −65, *d* = 2 for inhibitory neurons.

### 4.3. Trained readout

To generate a real-valued output from the discrete spiking activity, the spike train from each neuron is convolved with a decaying exponential according to Equation (5). The vector of values produced is then weighted with the readout weight matrix and summed to produce a single output value, shown in Equation (6).

(5) $$\begin{array}{rcl} x_{i} & = & f\left(S\left(t\right)\right)=max\left(\overset{T}{\underset{t=1}{\sum }}exp\left(\frac{-S\left(t\right)}{\tau }\right)\right) \end{array}$$

(6) $$\begin{array}{rcl} y & = & \overset{n}{\underset{i=1}{\sum }}x_{i}▪w_{i} \end{array}$$

The state vector for a neuron is denoted by *x*_*i*_, the filter function is *f*() and the spike train is *S*(*t*). The maximum number of time-steps in *S*(*t*) is *T*, in this case 50. The decay constant τ is 6 ms.

The maximum value is taken from the low-pass filtered values in Equation (5) in order to detect the highest level of burst activity in the given neuron. We take this approach under the assumption that burst activity is more representative of spiking neural computation than a sum total of the firing rate.

These output weights are updated according to the iterative, stochastic gradient descent method: Least Mean Squares, given in Equation (7).

(7) $$\begin{array}{r} w_{i}\leftarrow w_{i}+\mu \left(y_{d}-y_{o}\right)x_{i}. \end{array}$$

Here, *y*_*d*_ is the desired output, *y*_*o*_ is the actual output, *x*_*i*_ is the input taken from a neuron's filtered state, and μ is a small learning rate of 0.005. The weight from *x*_*i*_ to the output is *w*_*i*_. For the classification tasks of pattern recognition, *y*_*d*_ takes the values of 0 or 1 depending if the class corresponding to the readout is the label of the current input sample.

### 4.4. Synaptic plasticity models

Three synaptic plasticity mechanisms are employed in this study, each of them based on the Hebbian postulate (Hebb, 1949) of “neurons that fire together, wire together.” Each mechanism is outlined as follows:

#### 4.4.1. BCM plasticity

The BCM rule (Bienenstock et al., 1982) is a rate based Hebbian rule that also regulates the post-neuron firing rate to a desired level. It works on a temporal average of pre- and post-synaptic activity. The BCM rule is given in Equation (8). The regulating parameter is the dynamic threshold θ_*M*_, which changes based on the post-synaptic activity *y* in the following function: θ_*M*_ = *E*[*y*], where *E*[·] denotes a temporal average. In our case, *E*[·] is calculated as an exponential moving average of the post-synaptic neurons membrane potential. The exponential decay coefficient used for this is 0.935. As the membrane potential is model-dependant, we normalize it between 0..1 in real-time by continuously updating max and min variables of previous values. There is also a uniform decay parameter ϵ*w* set as 0.0001 that slowly reduces connection strength and so provides a means for weight decay, irrespective of the level of activity or correlation between pre-synaptic inputs and post synaptic potential. A plot of the BCM weight change is presented in Supplementary Figure 9.

(8) $$\begin{array}{l} \Delta w=y\left(y-\theta_{M}\right)x-\epsilon w \end{array}$$

#### 4.4.2. Bi-phasic STDP

The STDP rule depends on the temporal correlation between pre- and post-synaptic spikes. The synaptic weight change is computed based on the delay between the firing times of the pre- and post- neuron. This is described in a fixed “learning window” in which the y-axis is the level of weight change and the x-axis is the time delay between a pre- and post-synaptic spike occurrence. The bi-phasic STDP rule consists of two decaying exponential curves (Song et al., 2000), a positive one to potentiate in-order spikes, and a negative one to depress out-of-order spikes. This rule was derived from experimental work carried out on populations of neurons *in vitro* (Markram et al., 1997; Bi and Poo, 1998). Bi-phasic STDP is given in Equation (9).

(9) $$\Delta w(\Delta t)=\{\begin{array}{ll} A_{+}\cdot \text{exp}(\frac{-\Delta t}{\tau_{+}}) & \text{ if }t>0\\ -A_{-}\cdot \text{exp}(\frac{\Delta t}{\tau_{-}}) & \text{ if }t\leq 0 \end{array}$$

*A*_+_ and *A*_−_ are the learning rates for the potentiation and depression, respectively. Δ*t* is the delay of the post-synaptic spike occurring after the transmission of the pre-synaptic spike. τ_+_ and τ_−_ control the rates of the exponential decrease in plasticity across the learning window. For our experiments the learning window is symmetric with *A*_+_ = *A*_−_ = 0.15 and τ_+_ = τ_−_ = 20 ms.

#### 4.4.3. Tri-phasic STDP

A tri-phasic STDP learning window consists of a narrow potentiating region for closely correlated activity but depressing regions on either side: for recently uncorrelated activity, and for correlated but late activity. This learning window has been observed *in vitro*, most notably in the hippocampi, between areas CA3 and CA1 (Wittenberg and Wang, 2006). The tri-phasic STDP is given in Equation (10).

(10) $$\begin{array}{r} \Delta w\left(\Delta t\right)=A_{+}\exp\left(\frac{-{\left(\Delta t-15\right)}^{2}}{200}\right)-A_{-}\exp\left(\frac{-{\left(\Delta t-15\right)}^{2}}{2000}\right) \end{array}$$

The learning rates are set as *A*_+_ = 0.25 and *A*_−_ = 0.1. Both STDP learning windows are plotted in Supplementary Figure 10.

### 4.5. Synaptic interference measure

We wish to quantify interference directly between synaptic adaptations of plasticity. Our formulation of synaptic interference is based on the synaptic changes from sequentially presented samples. Synaptic adaptation for a given class of sample is called Δ*W*_*t*_ and average adaptation for all others are Δ*W*_*o*_. Interference must be calculated individually for each class of sample, $I_{t}^{class}$, and averaged together to get the overall interference, *I*^*total*^. The equations are as follows:

(11) $$\begin{array}{rcl} I_{t}^{\mathrm{class}} & = & \frac{1}{N}\overset{N}{\underset{i=1}{\sum }}\left[\Delta W_{ti}\cdot \Delta W_{oi}<0\right]\left[|\Delta W_{ti}|<|\Delta W_{oi}|\cdot C_{n}\right] \end{array}$$

(12) $$\begin{array}{rcl} I^{\mathrm{total}} & = & \overset{C_{n}}{\underset{t=1}{\sum }}\frac{I_{t}^{\mathrm{class}}}{C_{n}} \end{array}$$

Where *I* is interference, *N* is the number of synapses, *C*_*n*_ is the number of competing sample classes and Δ*W* is a vector of synaptic changes. Subscript *i* denotes the parameter index, subscript *t* denotes samples of a given class “this” and subscript *o* denotes samples of all “other” classes.

In Equation (11), the first set of Iverson brackets returns 1 if synaptic adaptation of a given class is of a different sign than that of the average adaptation of other class samples. The second set of Iverson Brackets returns 1 only if the magnitude of the synaptic adaptation of a class is less than the average weight adaptation of other classes multiplied by the total number. This leads to us taking a conservative measure of synaptic interference where we will only flag interference within a synapse for a class of pattern if the weight change is in a different direction to the average as well as being lower in magnitude than the total weight adaptation of other inputs.

### 4.6. Synthetic signal data

A synthetic benchmark task is taken from a study performed with Echo State Networks (Jaeger, 2007), a similar type of network model to the one we employ, but using continuous rate-based neurons instead. The task is to predict which of three signal generating functions is currently active in producing a time-varying input signal. To generate a sample of the signal at a given time step, one of the three following function types is used; (1) A sine function of a randomly selected period, (2) A chaotic iterated tent map, (3) A randomly chosen constant. The generator is given some low probability, 0.05, of switching to another function at each time-step. The full method of generating the data is described in Jaeger (2007). A short window of the generated signal is plotted in Figure 4.

### 4.7. Speaker recognition data

A speaker recognition task is a classification problem dealing with mapping time-series audio input data to target speaker labels. We use a data set taken from Kudo et al. (1999) which consists of utterances of nine male Japanese speakers pronouncing the vowel **/ae/**. The task is to correctly discriminate each speaker based on the speech samples. Each sample is comprised of a sequence of 12 feature audio frames. The features of each frame are the LPC cepstrum coefficients. The sample sequence ranges between 7 and 29 frames. The dataset is divided into training and testing sets of 270 and 370 samples each, respectively. Note that unlike the benchmark data used in this report, the samples are not in a consecutive time-series, yet each sample consists of a time-series sequence of audio frames.

### 4.8. Pre-processing of the human motion data

A visual task is selected to test high dimensional spatial-temporal input data. The KTH data set (Schuldt et al., 2004) consists of 2391 video files of people performing one of six actions; boxing, clapping, waving, walking and jogging. There are 25 different subjects and the samples cover a range of conditions that are described in more detail in Schuldt et al. (2004). Each video sample is taken at 25 frames per second and down sampled to a resolution of 160 × 120 pixels. We process the raw video sequences according to a formula shown in the following equations:

(13) $$\begin{array}{l} M\left(t\right)=∥\left[\Delta \left(I_{1},I_{2}\right),\ldots ,\Delta \left(I_{N-1},I_{N}\right)\right]∥ \end{array}$$

(14) $$M(t,i)=\{\begin{array}{ll} 1 & \text{ if }M(t,i)\geq 0.2\cdot max(M(\cdot ))\\ 0 & \text{ else} \end{array}$$

The final input matrix *M* is indexed by time-frames, *t* and spatial samples *i*. Column vectors *I*_*n*_ are individual frames, re-shaped into one dimension. Each sample contains up to a total of *N* frames. In plain language, this process essentially further down samples by a factor of 0.2 and calculates the difference between pixels in consecutive frames, which are then used as the new input features. Each frame is then re-shaped into a single dimensional column vector then appended together to form an input matrix in which each column is used as the neural network input at consecutive time steps. Figure 3 shows frames extracted from an example of each type on motion along with the corresponding processed features.

## Author contributions

Conception and design of the work was by YJ and JC. Experiments were performed by JC. Analysis and interpretation were undertaken by JC and YJ. Manuscript was written by JC and revised by YJ.

### Conflict of interest statement

The authors declare that the research was conducted in the absence of any commercial or financial relationships that could be construed as a potential conflict of interest.
