# Supplementary material for: Learning structure of sensory inputs with synaptic plasticity leads to interference
Source: Front Comput Neurosci. 2015 Aug 5;9:103. doi: 10.3389/fncom.2015.00103 (PMC4525052; doi:10.3389/fncom.2015.00103)
Supplement: Supplementary file 1 [file Image1.PDF]

Supplementary Material: Learning Structure of Sensory Inputs with Synaptic Plasticity Leads to Interference

Joseph Chrol-Cannon [1] **and Yaochu Jin** [1,*]

[1] *Department of Computing, University of Surrey, Guildford, UK*

Correspondence*:
Yaochu Jin
Department of Computing, University of Surrey, Guildford, GU2 7XH, UK,
yaochu.jin@surrey.ac.uk

1 SUPPLEMENTARY FIGURES

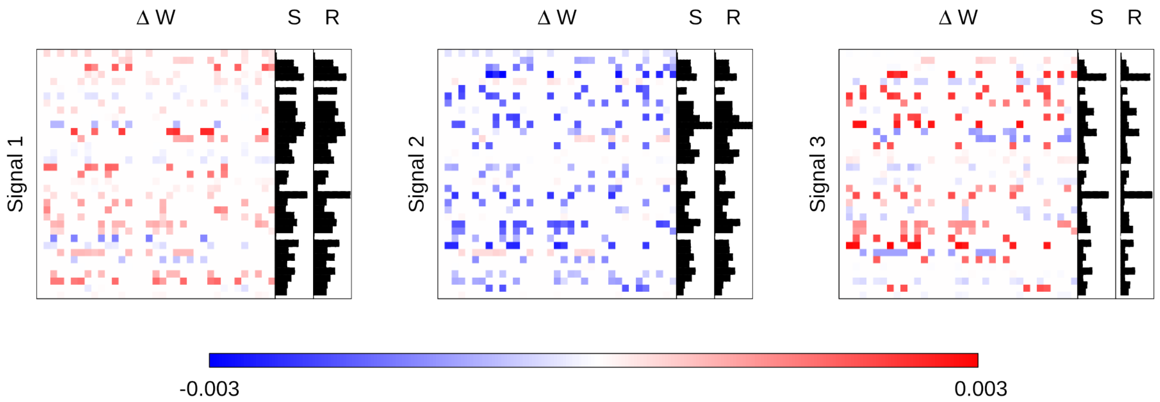

Supplementary Figure 1. The class-specific weight adaptation for the 3 class time-series benchmark task under BCM plasticity. Description of each sub-plot follows Figure 4 in the article.

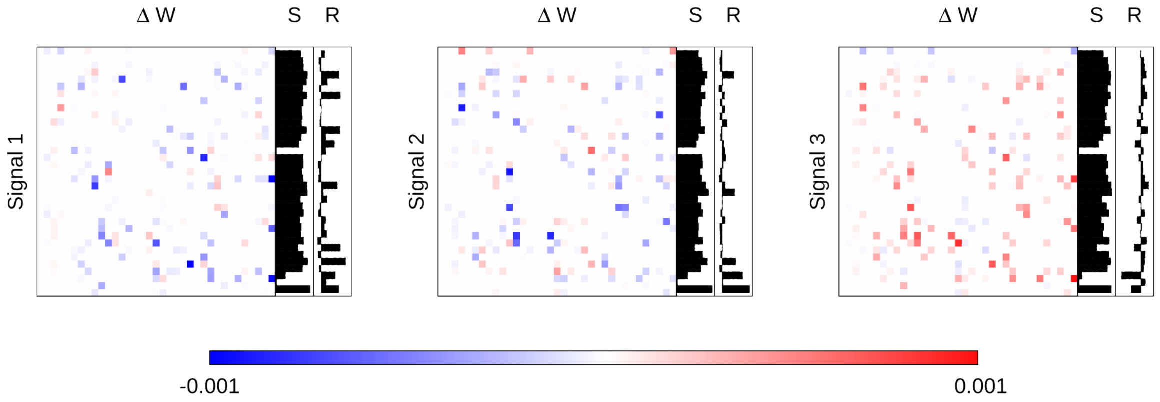

Supplementary Figure 2. The class-specific weight adaptation for the 3 class time-series benchmark task under bi-phasic STDP. Description of each sub-plot follows Figure 4 in the article.

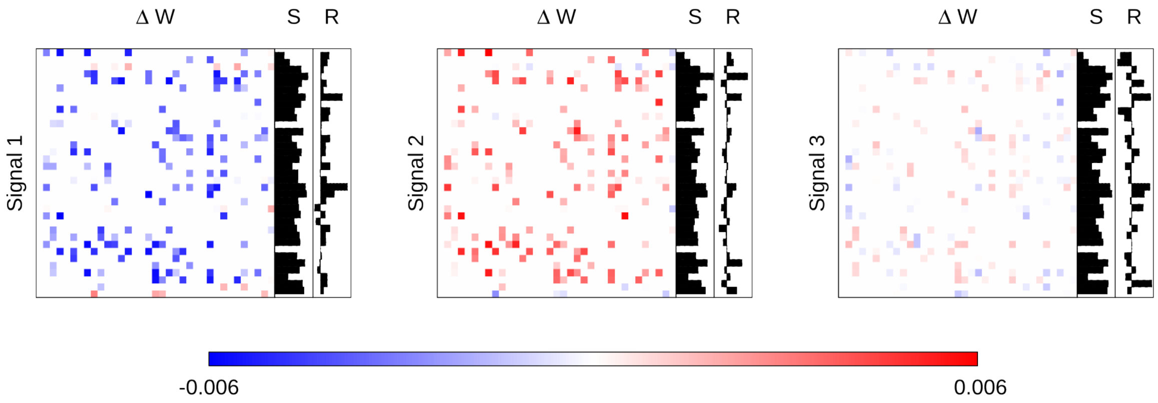

Supplementary Figure 3. The class-specific weight adaptation for the 3 class time-series benchmark task under tri-phasic STDP. Description of each sub-plot follows Figure 4 in the article.

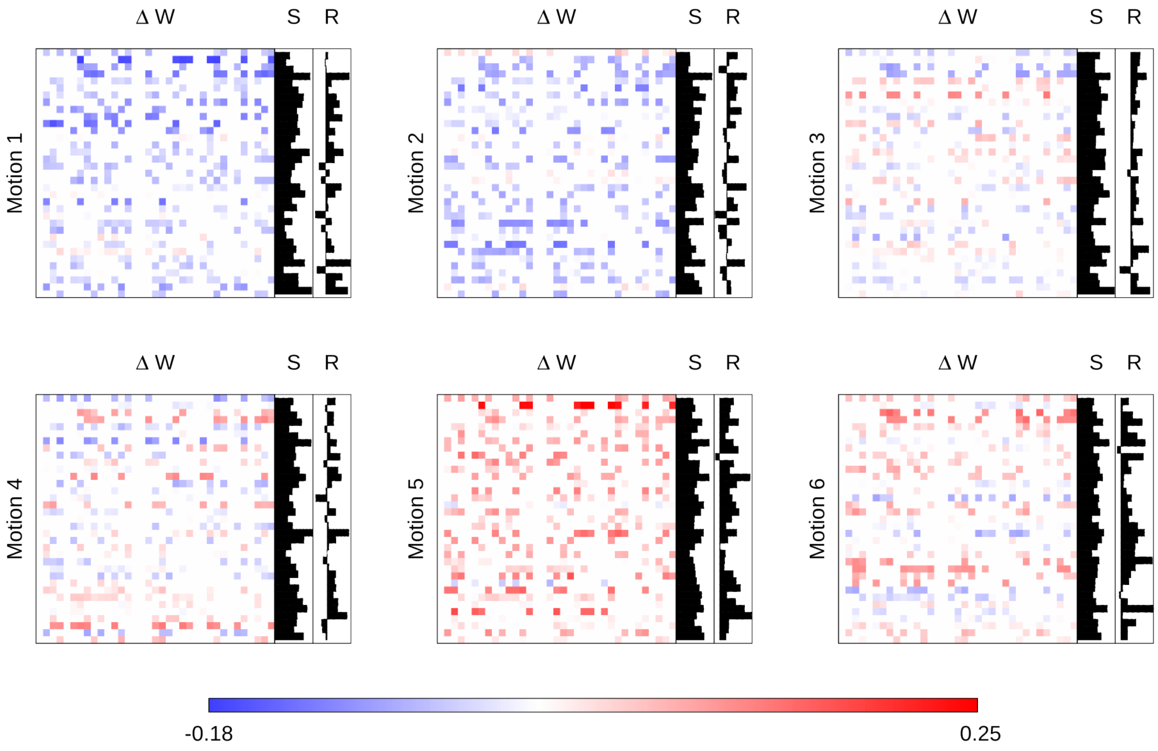

Supplementary Figure 4. The class-specific weight adaptation for the 6 class time-series human behaviour recognition task under BCM plasticity. Description of each sub-plot follows Figure 4 in the article.

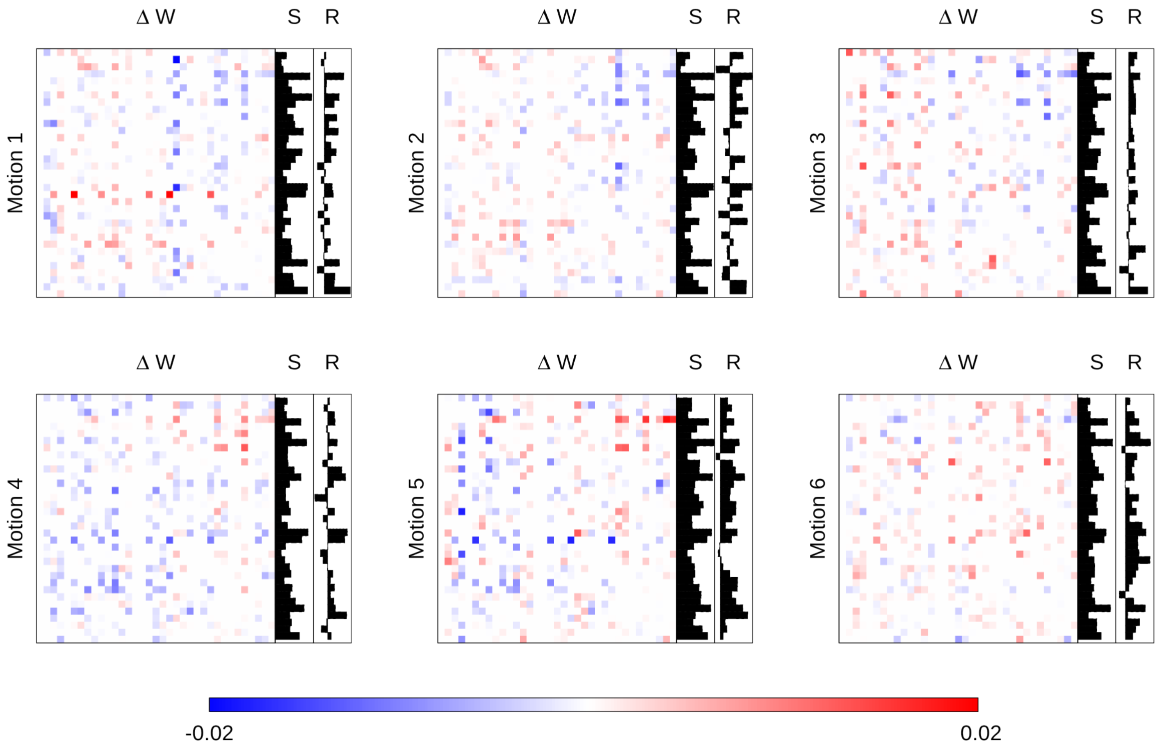

Supplementary Figure 5. The class-specific weight adaptation for the 6 class time-series human behaviour recognition task under bi-phasic STDP. Description of each sub-plot follows Figure 4 in the article.

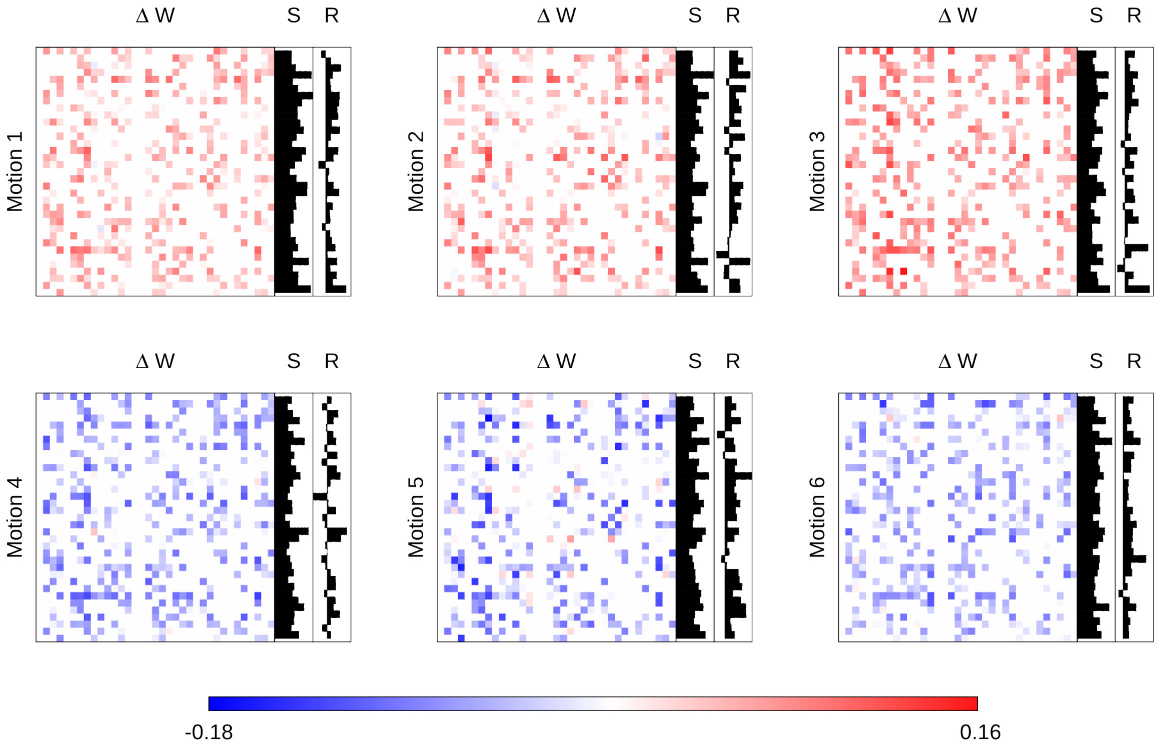

Supplementary Figure 6. The class-specific weight adaptation for the 6 class time-series human behaviour recognition task under tri-phasic STDP. Description of each sub-plot follows Figure 4 in the article.

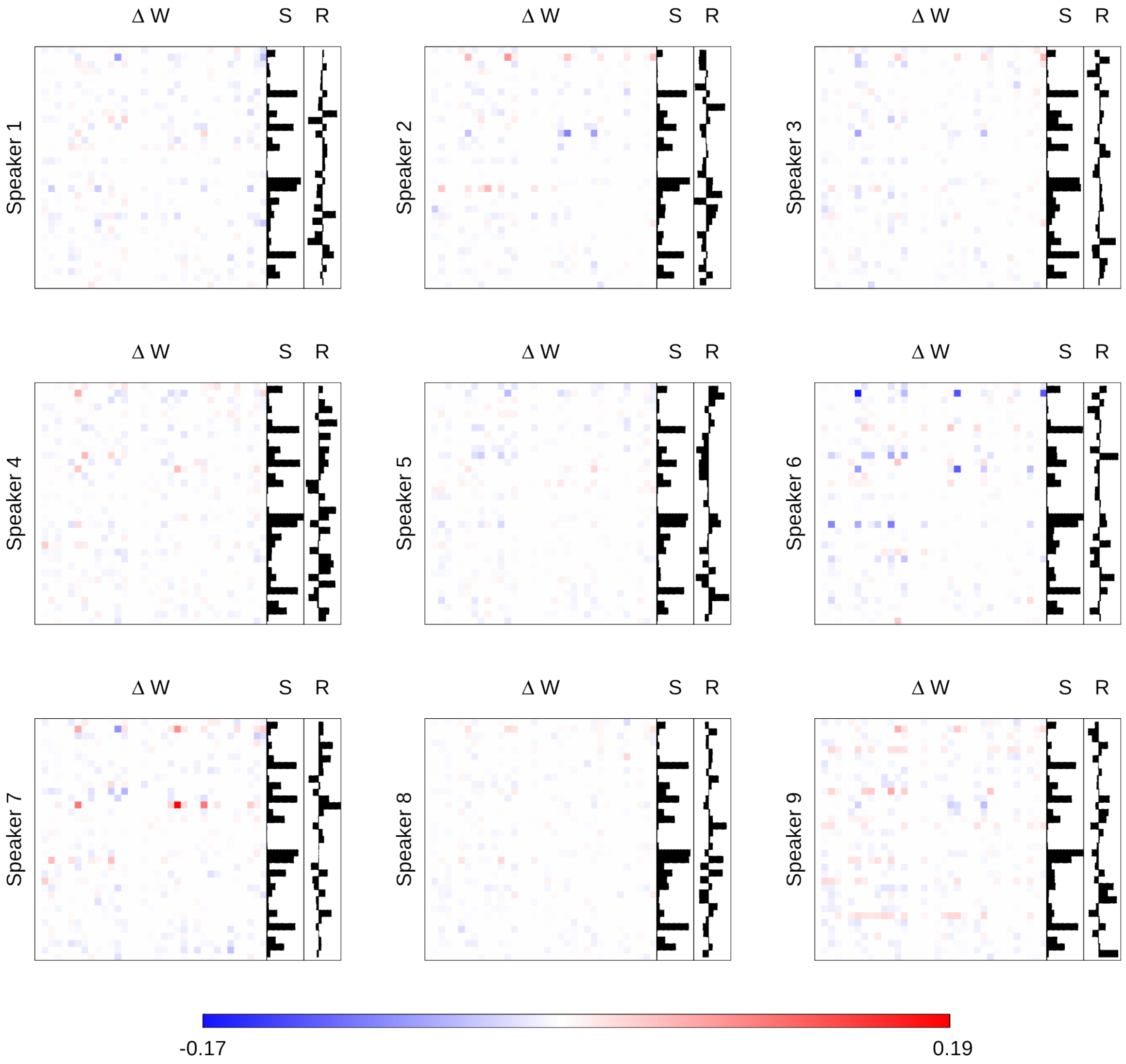

Supplementary Figure 7. The class-specific weight adaptation for the 9 class speaker recognition task under STDP. Description of each sub-plot follows Figure 4 in the article.

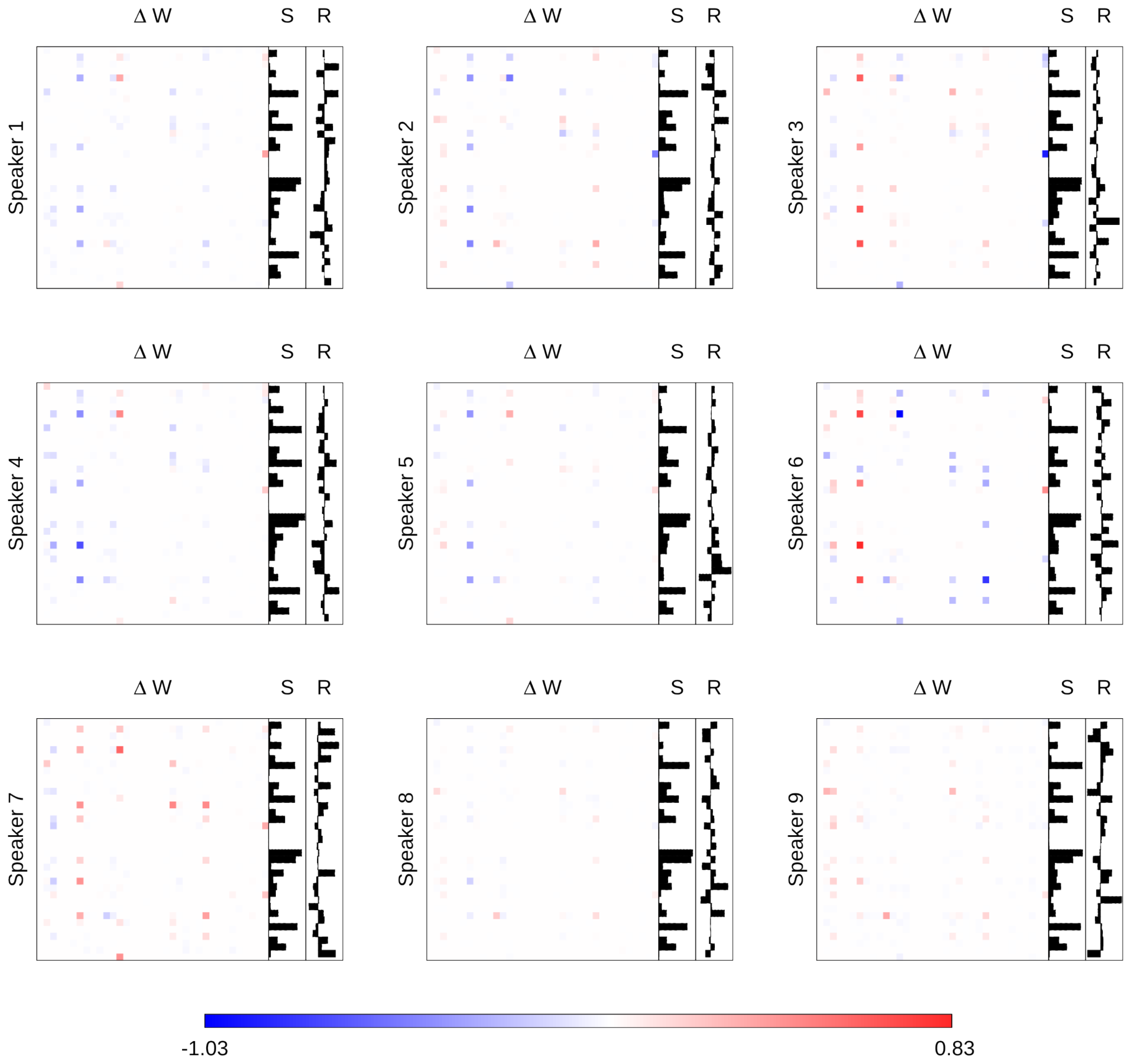

Supplementary Figure 8. The class-specific weight adaptation for the 9 class speaker recognition task under Tri-phasic STDP. Description of each sub-plot follows Figure 4 in the article.

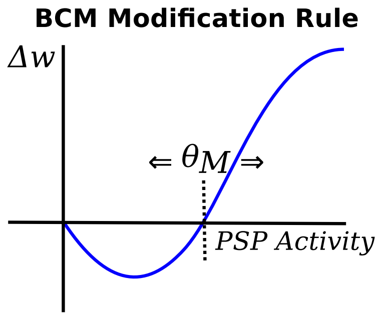

Supplementary Figure 9. The Bienenstock-Cooper-Munro plasticity rule illustrated with synaptic weight change on the y-scale and post-synaptic activity on the x-scale. θ_M is the sliding modification threshold that changes based on a temporal average of post-synaptic activity.

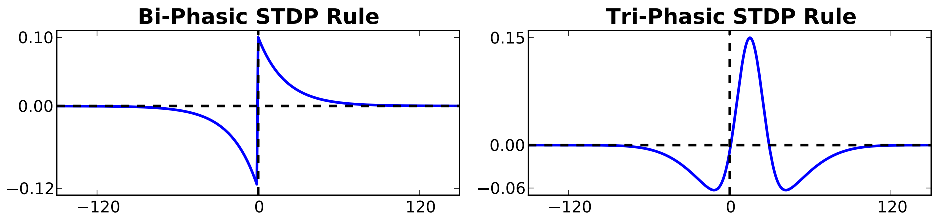

Supplementary Figure 10. The two predominantly studied STDP learning windows.
